# Supplementary material for: Chromothripsis during telomere crisis is independent of NHEJ, and consistent with a replicative origin
Source: Genome Res. 2019 May;29(5):737–49. doi: 10.1101/gr.240705.118 (PMC6499312; doi:10.1101/gr.240705.118)
Supplement: Supplemental Material [file supp_gr.240705.118_Supplemental_file_1.zip › contigs/annotated_contigs/DB71/contig.2.DB71_length_428_mean_cov_39.4976635514.docx]

**DB71_length_428_mean_cov_39.4976635514**

```
GCCCGCCTGTCTAGATTCCTCTCGGCCTCTCTAAGCACCGAAGCACGCGCTTCTCACTTTCGTGGAAGGGGCAGGGCCCTACCCGGCAC
 >chrUn_gl000220:129128-129489 - E=2e-176 p=0e+00
GGGGGTGTCTGACAGACTGACAGAGAAAGAGACAGACATAGAAAGACAGAGATGGACAGCGAGAGATAGAGAGAAACAGACAGACAGAG

AGAGAGAGAGAGAGAGAGAGAGAGAAACAGACAGGGAGGGAGAAAGACAAATAGAGAGAGAGACAGACAGACACACAGAGAGACAGACA

GAGAGAGAAACAGACAGAAAGAGAGAGAGACGGAGAGAGAGTGAGTGAGAGAGAGAGAGAGAGAGAGGGAGGGAGAGAGACAGACAGA|

GAGAGAGACAGAGAGAGAGAGAGAGAGAGAGGGAGAGAGAGAGAGAGAGAGAGGGGGG|GGGAAGGCGGGGAGA >chr8:143968815-143968873 + E=2e-14
```
